# The Treatment of Ulcers

**Published:** 1888-10

**Authors:** 


### The Treatment of Ulcers.

An article appeared in the *London Medical Record* of December 15, 1887, giving interesting details of the treatment of ulcers by phosphoric acid, as shown by the experience of Dr. Grossich. By this method of treatment, he used a ten per cent. solution of pure phosphoric acid in distilled water. The ulcer is covered with a bit of lint dipped in this solution, and the dressing renewed three or four times a day. The patient for the first few minutes feels a slight burning sensation, but this soon passes, and within twenty-four or thirty-six hours the ulcer cleans, and looks better. Inflammation or eczema of the surrounding parts disappears, and all pruritus ceases. The ulcer cicatrizes rapidly, and the cicatrix is firm and healthy.

Kollischer treated tubercular affections of the joints with injections of the phosphate of lime with great success. Dr. Grossich has also had good results with this treatment, and cites some very interesting successful cases.

The treatment by the solution of phosphoric acid was further employed in a case of tuberculous abscess of eight month's duration, and also a case of eczema marginatum which has lasted more than a year, and good results followed.

The above suggests a trial of Horsford's Acid Phosphate as a substitute for the phosphoric acid.

